# Peptide Nanotube Encapsulated Enzyme Biosensor for Vapor Phase Detection of Malathion, an Organophosphorus Compound

**DOI:** 10.3390/s19183856

**Published:** 2019-09-06

**Authors:** Christopher Edwards, Surachet Duanghathaipornsuk, Mark Goltz, Sushil Kanel, Dong-Shik Kim

**Affiliations:** 1 Department of Systems Engineering and Management, Air Force Institute of Technology (AFIT), WPAFB, OH 45433, USA (C.E.) (M.G.) (S.K.); 2 Department of Chemical Engineering, University of Toledo, Toledo, OH 43606, USA

**Keywords:** organophosphate vapor, peptide nanotube, butyrylcholinesterase, malathion, cyclic voltammetry, horseradish peroxidase

## Abstract

This study explores the use of a butyrylcholinesterase (BChE)-based, reversible reaction biosensor using screen-printed electrodes (SPEs) having a smaller working surface area than the single-use electrodes previously studied. Previous research demonstrated the prospective application of a single-use biosensor fabricated with an acetylcholinesterase (AChE) enzyme encapsulated in peptide nanotubes (PNTs) and enhanced with horseradish peroxidase (HRP) to detect organophosphorus compounds (OPCs) in aqueous and gas phases. In the current study, potential improvements to the biosensor are investigated. BChE-based biosensors were fabricated using PNTs, HRP, and Nafion in combination to increase the reactive surface area, enhance sensitivity, and maintain enzyme stability. Cyclic voltammetry (CV) was used along with the new modified sensor to measure malathion concentration in the gas phase. The results show that a BChE-based biosensor could reliably measure gas phase malathion concentrations between 6–25 ppbv by CV with the extent of inhibition linearly proportional to the malathion concentration (R^2^ = 0.941). This research demonstrated that fabricated BChE-based biosensors could be stored without cold storage requirement for up to six weeks with minimal performance degradation. Moreover, the sensor electrodes were each reused several times, and were still useable at the conclusion of the research. This research demonstrates the potential of fabricating a reusable, inexpensive biosensor that is capable of OPC detection with high sensitivity and a low detection limit without a long-term cold storage requirement.

## 1. Introduction

Organophosphorus compounds (OPCs) include insecticides (malathion, parathion, diazinon, and fenthion) [1], which irreversibly bind to acetylcholinesterase (AChE) receptors in the central nervous system. OPCs prevent the nervous system from hydrolyzing acetylcholine (ACh), which consequently builds up, and as a result, continuously stimulates muscles. When a vital muscle such as the diaphragm cannot relax, suffocation results and causes death within minutes [2]. Based upon the interaction of OPCs with AChE, previous studies have investigated use of esterase-based biosensors to detect OPCs in liquid [3,4,5] and gas phases [6]. Detection is based upon a redox reaction facilitated by the presence of AChE, using ACh or ASCh (acetylthiocholine) as substrates. OPCs inhibit the reaction, and the concentration of the OPC can be determined by using a cyclic voltammeter to measure the extent of inhibition. The AChE-based biosensor is fabricated on a gold screen-printed electrode (SPE) using peptide nanotubes (PNTs), Nafion, and horseradish peroxidase (HRP) in combination to increase the reactive surface area, enhance sensitivity, and preserve enzyme stability over time. Baker [6], who used acetylthiocholine (ASCh) as the substrate, demonstrated that the inhibition of the current response of the AChE/ASCh reaction by a gas phase OPC was proportional to the OPC concentration. Baker’s work was carried out on single-use, disposable SPEs, and closely paralleled a similar research effort by Arduini et al. [7], who also developed a disposable electrochemical biosensor with a shelf-life greater than six months. To overcome the irreversible covalent binding associated with AChE, another enzyme with demonstrable reversible binding was sought out and used.

Butyrylcholinesterase (BChE) is another enzyme that covalently reacted with OPCs as the inhibiting reaction and is considered as a biorecognition sensing element for OPCs [8,9]. Several researchers made use of the inhibition reaction of BChE with OPCs to detect the presence of nerve agents and pesticides. For example, Arduini et al. [10] demonstrated the cost-effective application of a Prussian blue-modified silver SPE using a BChE enzyme and butyrylthiocholine (BSCh) as the substrate to detect the gas phase OPC nerve agents, paraoxon, sarin, and VX [10]. Campanalla et al. [11] also used BChE in an oxygen amperometric transducer to detect both organophosphorus and carbamate pesticides in water-saturated chloroform medium. They successfully detected both nerve agents and pesticides at the low detection limit of the order of 10^−5^–10^−6^ mmol/L. Since BChE has a higher inhibiting reaction and sensitivity to some OPCs (e.g., paraoxon and heptenophos) [10,12] with better stability on a sensor surface compared to AChE [13], we investigated the potential of fabricating a BChE-based biosensor for detecting the OPC malathion in gas phase on a reusable gold-SPE, modified with PNT, HRP, and Nafion.

**Figure 1:**
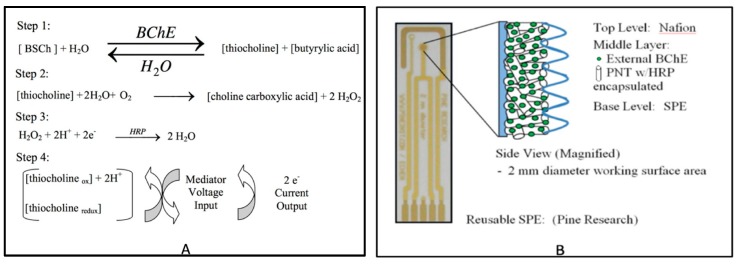
(**A**) Butyrylcholinesterase/butyrylthiocholine (BChE/BSCh) enzyme electrochemical reaction-based biosensor (Adapted from Andreescu, 2006) [15]; (**B**) Biosensor Construct.

The principle upon which a BChE-based biosensor works is depicted in Figure 1A [14,15]. BChE catalyzes the hydrolysis of BSCh (Step 1). Step 1 of the reaction is inhibited by the presence of an OPC, because the OPC binds with the BChE enzyme. The intermediate breakdown of thiocholine in Step 2 produces hydrogen peroxide. Hydrogen peroxide is hydrolyzed in the presence of the HRP catalyst, and then is broken down into water at Step 3. Two moles of electrons generated in these redox reactions are transduced to the gold electrode and represented as the current change in cyclic voltammetry. In the presence of OPC, BChE is inhibited by it, and the electrical current is reduced, depending on the concentration of OPC. The reduction of current due to the BChE inhibition by OPC is equivalent to the OPC concentration bound to the BChE on the SPE surface. HRP serves as a catalyst during Step 3. Thus, the addition of HRP on the sensor increases the sensitivity of the sensor and allows the reaction to take place at lower voltages [6] which, in turn, reduces the interference signals from the background current [16].

Peptide nanotubes (PNTs) have been used to enhance enzyme function and enzyme stability in biosensors [17]. The PNTs serve to protect enzyme activity [18], thereby increasing the shelf-life and performance of the biosensor. Biosensor fabrication may also include the application of a final top-layer component. The top layer provides additional protection as well as acting as an adhesive to bind the PNTs to the electrode. Nafion, a Teflon-based stable polymer, has been applied to bind PNTs and their associated enzymes to an electrode [19]. When used in combination, the three materials, PNTs, HRP, and Nafion, are used to increase the contact area between the enzymes and the chemical compounds, protect the enzymes, and increase sensor sensitivity, as shown in Figure 1B.

The goal of this line of research is to create an inexpensive BChE-based biosensor that is capable of OPC detection and explore its performance characteristics: longevity, i.e., post-fabrication shelf-life under laboratory conditions, sensitivity in terms of limit of detection (LOD), precision, OPC concentration–inhibition response relationship, and reusability of the SPE when using commercially available off the shelf (COTS) reusable SPEs.

## 2. Results and Discussion

### 2.1. The Investigation of Electrode Configuration

#### 2.1.1. Different Layering Configurations of Biosensors

**Figure 2:**
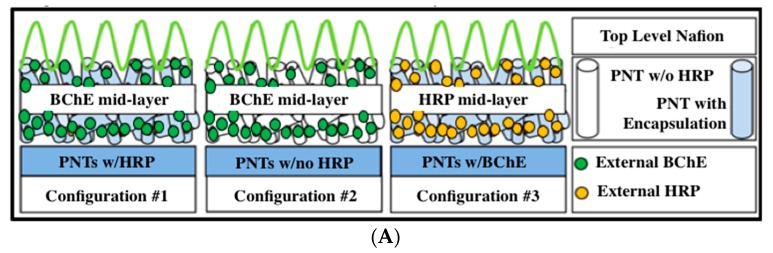

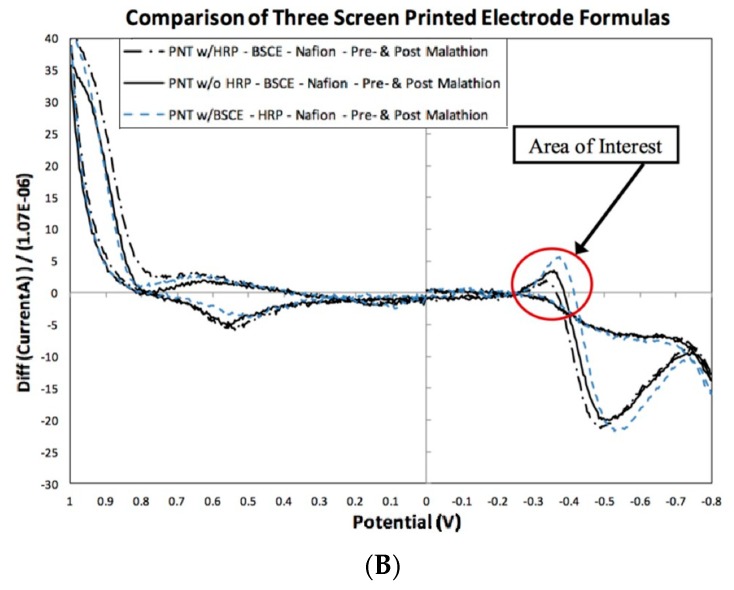
(**A**) Three different sensor configurations; (**B**) Cyclic voltammogram of three different configurations for malathion inhibition.

Prior to committing resources to sensitivity and longevity research, three different configurations of PNT, HRP, BChE, and Nafion were prepared and investigated to decide on the most promising sensor configuration to research for malathion detection. In these initial sensor system configurations, BChE was used as the reversible sensing enzyme to bind with malathion. The choice of electrode configurations was based on the fabrication options and resources available. Configuration #1 was prepared by having PNTs encapsulating HRP as the first layer, followed by BChE application and then Nafion. Configuration #2 was prepared using PNTs without HRP encapsulation with BChE then added, and finally Nafion. Lastly, Configuration #3 was prepared with PNTs encapsulating BChE; then, HRP was added to this middle layer, followed by Nafion on the top layer. Three different configurations are shown in Figure 2A. Cyclic voltammetry was used to investigate the sensing potential of three different configurations with constant 25 ppbv malathion concentration. Changing the position of BChE and HRP explored how the configuration change may produce different results. In the presence of butyrylthiocholine (BSCh), the BChE enzyme enables BSCh conversion to thiocholine. Then, thiocholine is further oxidized to choline carboxylic acid and hydrogen peroxide, which is reduced to water via HRP. The two moles of electrons are produced in Step 3, and detected with the electrode as the electrical signal. When OPC is present, this reaction scheme is disturbed due to the inhibition of BChE by OPC, and as a result, the electrical current will reduce, depending on the OPC concentration. Therefore, a measured change of the electrical signal represents the OPC concentration. For this exploratory test, two electrodes were prepared for each configuration and tested. The sensing potential was measured from the area of potential interest (−0.30 V through −0.40 V), which is referred to the percentage of malathion inhibition, as shown in Figure 2B and Table 1. Each curve in Figure 2B represents the difference between the currents of “pre-malathion exposure” and “post-malathion exposure” of a sensor. For all configurations, the greatest current change occurred at −0.30 to −0.40 V, as shown by the blue dotted line.

Based on Figure 2B and Table 1, the highest malathion inhibition achieved was Configuration #3: PNT with encapsulated BChE, followed by HRP application, and Nafion added as a protective cover. Configuration #3 notably achieved the highest inhibition percent and successfully utilized the PNT encapsulation, HRP enzyme, and Nafion protection to measurably increase the sensor inhibition capability. It is thought that in Configuration #3, most of the H_2_O_2_ and two moles of electrons generated via BChE inside the PNT diffuse out of the PNT and then convert to H_2_O by HRP outside the PNT. Therefore, when malathion was present, the reduction of electrons due to the inhibition of BChE by malathion was observed with high inhibition percentages. On the other hand, in Configuration #1, much of the H_2_O_2_ and electrons generated via BChE outside the PNT diffuse out rather than diffusing inside the PNT and reducing to H_2_O. Therefore, the changes in the electrical signal in Configuration #3 showed greater inhibition results, even though less malathion may have been inhibited by BChE due to the mass transfer limit than in Configuration #1. However, the final selection criteria was based on the combination of a greater potential (V) range where the measured inhibition peak occurred, and the variation in the inhibition percent calculated for the two electrodes tested was the smallest. Configuration #1 was ultimately selected for further sensitivity and longevity study.

Note: Since this configuration test involved only two electrodes, the midpoint between two numbers rather than a statistical mean is shown. Also noted is that while both Configurations #2 and #3 achieved a higher inhibition percent during this exploratory test than Configuration #1, the inhibition percent measured for 25 ppbv malathion concentration was later statistically averaged at 65.68%.

#### 2.1.2. The Effect of Nafion Layer on the Electrode Efficiency

A Nafion layer was applied on top of the sensor to protect enzymes from degradation due to temperature, pH, and humidity for maintaining the enzyme stability. For this next experiment, Configuration #1 was selected. Two sets of electrodes were prepared by having PNTs encapsulating HRP with BChE on top of the PNTs with and without a Nafion layer in the presence of 25 ppbv malathion concentration. As shown in Table 2, electrodes with a Nafion layer had a higher percentage of malathion inhibition compared to the electrodes without a Nafion layer. Moreover, it showed a better consistency for malathion inhibition with a standard deviation of 2.52 compared to 12.3 of the electrodes without a Nafion layer. Thus, this experiment confirmed the necessity of a Nafion layer for the enzyme-based sensor to improve the sensor efficiency and maintain the sensor consistency.

### 2.2. Biosensor Detection

#### 2.2.1. Detection of Malathion

**Figure 3:**
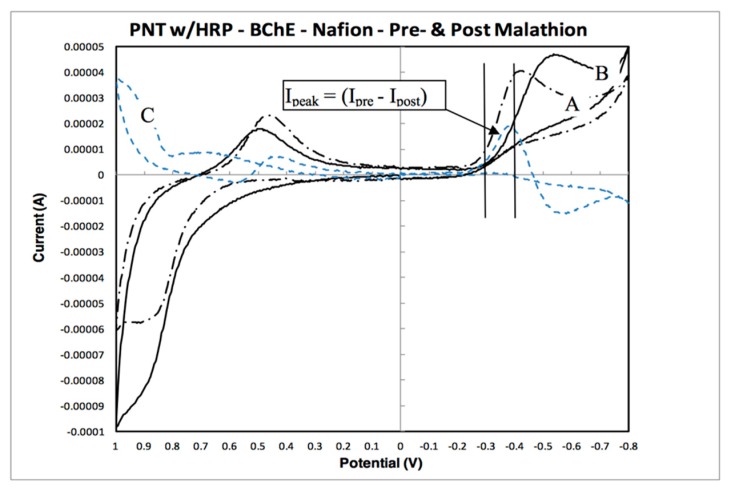
Representative CV data for peptide nanotubes (PNT) with horseradish peroxidase (HRP)/BChE/Nafion composition matrix: Line A is (I_pre_), line B is (I_post_), and line C is (I_pre_ − I_post_).

The analysis of cyclic voltammetry (CV) signatures for the BChE-based biosensor notably used the difference between pre-malathion and post-malathion exposures (Figure 3, lines A and B). The lines in Figure 3 are representative of a single biosensor electrode test. Line A represents a single scan of the fabricated biosensor electrode prior to malathion exposure for CV measurement number two (CV#2). Line B represents a post-malathion exposure scan, CV#4. Line C is the difference in current between lines A and B. The current change, ΔA, at −0.30 through −0.40 V the same area of interest shown in Figure 2—was used for the percent inhibition calculation. As shown in Figure 3, ΔA for malathion, the line C peak, which represents the current change between pre-malathion and post-malathion exposures, is the highest in the same area of interest, as indicated in Figure 2.

As shown in Figure 3, this fabricated BChE-based biosensor example has a line C “fingerprint” region from −0.30 through −0.40 volts, where a distinctive peak for line C is realized and reproducible. Thus, the line C peak was used to calculate the inhibition for each malathion concentration, as described below. For each biosensor tested, the percent inhibition was calculated using the following equation:

(1) $$\mathrm{Percent}\;\mathrm{Inhibition}=\frac{\left(I_{pre}-I_{post}\right)}{I_{pre}}\times 100$$

where *I_pre_* and *I_post_* are current values at a particular potential (V) before and after malathion exposure, respectively. As noted above, line C is the numerator in Equation (1). Thus, for each biosensor test, a peak in line C was located within the potential range −0.30 and −0.40 volts, and Equation (2) was applied:

(2) $$\mathrm{Percent}\;\mathrm{Inhibition}=\frac{{\left(I_{peak}\right)}_{line\;C}}{{\left(I_{pre}\right)}_{line\;A}}\times 100$$

The (*I_peak_*)*_line C_* is the current at the peak of line C (within the specified potential range). The (*I_pre_*)*_line_*_*A*_ is the current value on line A determined from the same voltage input as line C’s current peak output.

**Figure 4:**
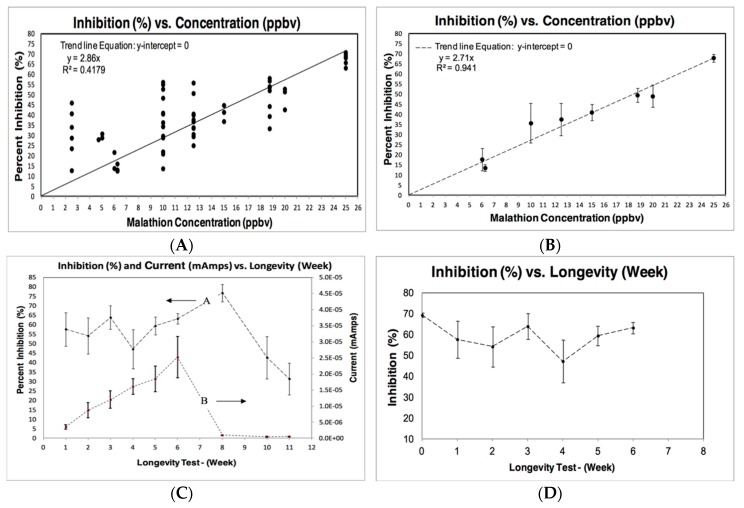
Biosensor test data results: (**A**) Malathion concentration from 0 ppbv to 25 ppbv, (**B**) Malathion concentration from 6 ppbv to 25 ppbv. BChE/BSCh Longevity Test: (**C**) Inhibition measured after biosensor exposure to 25 ppbv malathion, (**D**) First six weeks after biosensor exposure to 25 ppbv malathion.

To calculate an average percent inhibition from a particular CV scan, multiple pairs of data points along lines A and C (each pair of points corresponding to a particular voltage) were used in Equation (2) to calculate a percent inhibition at that voltage. Each data point shown in Figure 4A represents the statistical mean percent inhibition for a biosensor test. During the biosensor tests, a limited number of CV data tests failed to show the Figure 3 characteristic “fingerprint.” In these cases, the biosensor test data was invalidated and excluded from Figure 4A.

#### 2.2.2. Biosensor Test Data Results

Figure 4A,B show results for percent inhibition when only concentration is varied. All the results provided in Figure 4 represent Configuration #1 from Figure 2A. In Figure 4A, the linear correlation between percent inhibition and malathion concentration is relatively low, using the whole range of data collected for malathion concentration from 0 ppbv to 25 ppbv. Looking at Figure 4A, it appears that this low correlation may be due to the measurements made at malathion concentrations less than 6 ppbv. To explore this possibility, data collection below 6 ppbv was removed to create Figure 4B. To simplify Figure 4B, the data were reduced to a single pooled average for fixed malathion concentrations and plotted using a pooled standard deviation as “whiskers” for the experimental results. The response to a range of malathion concentration from 6 ppbv to 25 ppbv, as seen in Figure 4B, appears linear, and is markedly improved in comparison to Figure 4A. When we determined the biosensor’s malathion limit of detection (LOD) as 6 ppbv, we conclude that there is a linear relation with R^2^ = 0.94 between percent inhibition and malathion concentration for the range of concentrations between this LOD and the vapor pressure of malathion (25 ppbv).

### 2.3. Longevity Experiment

In Figure 4C, the relationship between the biosensor current and the percent inhibition is shown for all the data of the longevity experiment. For this experiment, Configuration #1 from Figure 2A was used. The “whiskers” in the figure represent the standard deviation for the weekly-calculated data point averages. The longevity test data indicate that both the biosensor current and percent inhibition should be considered when determining sensor shelf-life. The data show increased current response for malathion detection during the first six weeks. When the current response markedly decreases to nearly zero on all the data collected after week six, this decrease indicates rapid degradation of the sensor. This sensor degradation over time also occurred in the Baker et al. [6] research for the AChE-based biosensor. In Baker’s research, the performance of an AChE-based biosensor degraded significantly between 45–60 days of dry storage at 4 °C. In a replot of the percent inhibition data, Figure 4D removes the measured current (mAmps) data and data collection for weeks 8, 10, and 11. The remaining data indicate the percent inhibition with the malathion concentration for the first six weeks. The “whiskers” again represent standard deviation. This longevity test demonstrated that sensors could be fabricated and stored at room temperature for several weeks and be reliably used.

## 3. Materials and Methods

### 3.1. Materials

S-butyrylthiocholine chloride, butyrylcholinesterase from equine serum, cellulose acetate, 39.8 wt% acetyl content, ASCh, and H–Phe–Phe–OH were all purchased from Sigma Aldrich (St. Louis, MO, USA); malathion, horseradish peroxidase (HRP), and 1,1,1,3,3,3-hexafluoro-2-propanol were purchased from Sigma Aldrich (Milwaukee, WI, USA); Nafion 117 solution (approximately 5%), ammonium acetate, and potassium phosphate dibasic were purchased from Sigma Aldrich (Allentown, PA, USA). Deionized water was generated in the lab via reverse osmosis. Gold screen-printed electrodes (SPEs), model RRPE2001AU-6, with a 2-mm diameter gold working electrode, an Ag/AgCl reference electrode, a platinum counter electrode printed on a ceramic substrate, the electrode–potentiostat interface cable, and the jacketed compact voltammetry cell were all purchased from Pine Research Instrumentation (Durham, NC, USA). All electrochemical measurements were conducted using a Parstat 2273 Advanced Electrochemical System and Power Suite Software from Princeton Applied Research. The SPE media were dried using Air Force Institute of Technology (AFIT)-supplied nitrogen gas. Peptide nanotubes (PNTs) were agitated using a Cole-Parmer 8890 Sonicator. Experiments were completed using a Pine Research Instrumentation jacketed compact voltammetry cell. Malathion concentration was determined using an Agilent Technologies 6890N Network GC Systems model gas chromatograph mass spectrometer (GC/MS) [6].

### 3.2. Methods

#### 3.2.1. Synthesis of PNTs and the Encapsulation of BChE Inside the PNTs

PNTs were synthesized by dissolving 100 mg of H–Phe–Phe–OH in 1 mL of 1,1,1,3,3,3-hexafluoro-2-propanol. This mixture was swirled gently for a few seconds and then placed in a sonicator for five minutes to ensure complete dissolution. After that, 1 mL of PNT solution was dried overnight in a vacuum oven or ventilation hood with nitrogen gas applied. Then, 1 mL of 50 mM, 7.4 pH phosphate buffer solution (PBS) containing 1 mL of BChE was added to the PNT solution. The PNT mixture was vortexed briefly and then incubated on a rotator in a temperature-controlled environment at 5 °C, 30 rpm for one week. The PNT mixtures were kept refrigerated until needed for biosensor fabrication.

#### 3.2.2. Biosensor Fabrication and Reconstitution

As an initial test of a reusable electrode, the biosensors were prepared by first developing the PNT mixture. The PNT mixture was crafted prior to its anticipated need. PNT mixture fabrication included being vortexed briefly and then incubated on a rotator in a temperature-controlled environment at 5 °C, 30 rpm for one week. The PNT mixtures were kept refrigerated until needed for biosensor fabrication. The biosensors were prepared by first depositing 2.5 μL of PNTs containing the encapsulated BChE on the working electrode, which was then allowed to dry in a hood at room temperature and pressure (average of 65 °F and 745 mmHg). Then, 2.5 μL of 1000 U/mL HRP was deposited on top of the PNTs and allowed to dry. Next, 2.5 μL of Nafion was deposited and allowed to dry. Prior to selection and formal investigation into the compositional matrix described, several fabrication processes were tested. While some configurations involved a simple change in the layering order, or no protective Nafion covering, each compositional matrix was tested with the same protocol. Qualitative performance criteria were used to select the compositional matrix with the highest prospects for continued research. Ultimately, the primary test Configuration #1 (see Figure 2A) was chosen for this research; it utilizes PNT encapsulated BChE followed by HRP then Nafion, as described above.

After each CV experiment, the SPEs were initially cleaned using a methanol rinse. The CV instrument was subsequently utilized with the SPE immersed in a dilute acetic acid solution to further clean the SPE until the CV “fingerprint” plot demonstrated a baseline signature. The PNT mixture was vortexed briefly and then incubated on a rotator in a temperature-controlled environment at 5 °C, 30 rpm for one week. The PNT mixtures were kept refrigerated until needed for biosensor fabrication. The biosensors were reconstituted using the method initially described.

#### 3.2.3. Cyclic Voltammogram

Each CV test started and ended at zero volts potential. Beginning at zero volts potential, voltage first increased to one volt at a rate of 50 mV/s for 20 s, and then decreased at a negative rate of 50 mV/s until −0.8 volts potential was reached for 36 s. Finally, from −0.8 volts, the CV test would return to zero volts, taking 16 s. CV measurement number one (CV#1) verified the initial condition of the electrode prior to conducting the experiment. CV#1 and CV#3 are not shown in the figures, because they were used solely for qualitative purposes. However, data from CV#2 and CV#4 were used for the calculation of percent inhibition as previously described, and are shown in Figure 3. CV#3 provided the minimum control exposure time to malathion in the gas phase of 216 s. Similar to CV#1, CV#3 also verified the electrode condition while conducting the experiment. No CV#1 or CV#3 data were used for calculating percent inhibition. As each CV cycle took 72 s to complete, data was saved to a file at a rate of 14 samples per second. Each CV test consisted of three consecutive CV cycles and was completed after 3 min and 36 s.

#### 3.2.4. Malathion Detection

Vapor concentration for the sensitivity tests was adjusted by injecting a known volume of gas saturated at room temperature with malathion (vapor pressure = 25 ppbv) into a vial purged with nitrogen at constant temperature. After the malathion injection, the 40-mL vial would equilibrate for a minimum of one hour prior to CV test use. The same set of SPEs was used for every subsequent sensitivity experiment. After each sensitivity experiment at a specific malathion concentration, the SPEs were initially cleaned using methanol. The CV instrument was utilized with the SPE immersed in a dilute acetic acid solution to further clean the SPE until the CV “fingerprint” plot demonstrated a baseline signature. To evaluate SPE reusability, for each sensitivity experiment, the same SPEs were refabricated with new enzyme layering prior to exposure to a different malathion concentration. The test protocol first involved electrode placement into a CV flask filled with a 7.4 pH PBS. After electrode verification (indicated by a normal CV scan), the electrodes were inserted into a 20-mL CV flask containing a 1 mM concentration of BSCh with PBS solvent, and CV measurement number two (CV#2) was taken. Then, the electrodes were transferred to the 40-mL vial that had been previously prepared as described above, and CV measurement number three (CV#3) was taken. Finally, the biosensor was reinserted into the BSCh-containing vial to obtain a post-malathion CV measurement four (CV#4). The time between CV#1–4 measurements was kept to a minimum, as each biosensor was individually tested. Manual transfer time variation between CV#3 and CV#4 appeared to coincide with variation in the statistical standard deviation of percent inhibition calculated for individual sets of SPEs tested. BChE demonstrates a different affinity to OPCs than AChE [7,12]. It is thought that a weaker covalent bond allows malathion disassociation from the SPE between the time after CV#3, removal from the malathion vial, and CV#4 when the post-malathion measurement was taken.

#### 3.2.5. Longevity Experiment

Weekly longevity experiments involved the simultaneous preparation of several sets of biosensors that were fabricated and stored dry at room temperature in a dark cabinet. This research explored the possibility of fabricating SPEs without the need for a subsequent cold storage requirement. Furthermore, while the laboratory was air conditioned, strict temperature control was neither managed nor monitored. To test the robustness of the biosensor fabrication, all SPEs experienced both temperature and humidity environmental fluctuations associated with a normal and comfortably designed laboratory. At weekly intervals, one set of sensors underwent the same test protocol as described in the malathion detection section, except the OPC concentration remained constant at 25 ppbv malathion.

## 4. Conclusions

The current study demonstrated that BChE/BSCh biosensors can be constructed to detect gas phase concentrations of malathion well below its normal vapor pressure. Regarding the sensitivity tests, OPC detection using a BChE-based biosensor was demonstrably improved compared to Baker’s AChE-based biosensor [6]. At 6 ppbv, the LOD was lower than the LOD of 12 ppbv achieved with the AChE-based sensor. Increased sensitivity was achieved despite a reduction of the reusable electrode’s working surface area; it was one-fourth the size of the disposable electrode used for the AChE studies. Based on the percent inhibition of the BSCh hydrolysis reaction, quantitative measurements can be made for the malathion concentration between the LOD of 6 ppbv and the vapor pressure of 25 ppbv. The sensors can be fabricated and stored at room temperature for up to six weeks with minimal performance degradation. The overall performance of the BChE-based malathion detector with a gas-phase LOD of 6 ppbv developed in this study provides evidence that such a detector may have potential applications for chemical warfare agent detection.

The views expressed in this article are those of the author(s) and do not reflect the official policy or position of the acknowledged agency, organization, the United States Air Force, the Department of Defense, or the United States Government.

## Figures and Tables

**Table 1:** Average percentage of malathion inhibition of three different configurations.

Configuration #	Potential (V)	Inhibition (%)	Average
1	−0.30 through −0.32	39.41%, 41.11%	40.18%
2	−0.30 through −0.34	39.44%, 51.51%	45.28%
3	−0.34	39.41%, 57.18%	50.95%

**Table 2:** Average percentage of malathion inhibition of Configuration #1 with Nafion.

Electrode #	Inhibition (%)	Electrode #	Inhibition (%)
1 (With Nafion)	68.16	4 (Without Nafion)	53.61
2 (With Nafion)	65.74	5 (Without Nafion)	32.39
3 (With Nafion)	63.13	6 (Without Nafion)	38.04
Peak Average	65.68	Peak Average	34.03
(Std Dev)	(2.52)	(Std Dev)	(12.3)
